# Simultaneous induction and blockade of autophagy by a single agent

**DOI:** 10.1038/s41419-018-0383-6

**Published:** 2018-03-02

**Authors:** Karolina Kucharewicz, Magdalena Dudkowska, Anna Zawadzka, Mikolaj Ogrodnik, Andrzej A. Szczepankiewicz, Zbigniew Czarnocki, Ewa Sikora

**Affiliations:** 1 Laboratory of Molecular Bases of Aging, Nencki Institute of Experimental Biology, Polish Academy of Sciences, 3 Pasteur Street, 02-093 Warsaw, Poland; 2 Laboratory of Natural Products Chemistry, Faculty of Chemistry, University of Warsaw, 1 Pasteur Street, 02-093 Warsaw, Poland; 3 Laboratory of Molecular and Systemic Neuromorphology, Nencki Institute of Experimental Biology, Polish Academy of Sciences, 3 Pasteur Street, 02-093 Warsaw, Poland; 4 Present Address: Newcastle University Institute for Ageing, Institute for Cell and Molecular Biosciences, Campus for Ageing and Vitality, Newcastle University, Newcastle upon Tyne, NE4 5PL UK

## Abstract

Besides cell death, autophagy and cell senescence are the main outcomes of anticancer treatment. We demonstrate that tacrine-melatonin heterodimer C10, a potent anti-Alzheimer’s disease drug, has an antiproliferative effect on MCF-7 breast cancer cells. The main cell response to a 24 h-treatment with C10 was autophagy enhancement accompanied by inhibition of mTOR and AKT pathways. Significantly increased autophagy markers, such as LC3B- and ATG16L-positive vesicles, confirmed autophagy induction by C10. However, analysis of autophagic flux using mCherry-GFP-LC3B construct revealed inhibition of autophagy by C10 at the late-stage. Moreover, electron microscopy and analysis of colocalization of LC3B and LAMP-1 proteins provided evidence of autophagosome-lysosome fusion with concomitant inhibition of autolysosomal degradation function. After transient treatment with IC_50_ dose of C10 followed by cell culture without the drug, 20% of MCF-7 cells displayed markers of senescence. On the other hand, permanent cell treatment with C10 resulted in massive cell death on the 5th or 6th day. Recently, an approach whereby autophagy is induced by one compound and simultaneously blocked by the use of another one has been proposed as a novel anticancer strategy. We demonstrate that the same effect may be achieved using a single agent, C10. Our findings offer a new, promising strategy for anticancer treatment.

## Introduction

Cancer cells often become resistant to apoptotic death and thus, recently, much attention has been paid to the induction of cell senescence and/or autophagy as alternative targets of anticancer therapy^1,2^.

Senescent cells are irreversibly arrested in the cell cycle but they remain metabolically active. There are three types of cellular senescence—replicative one, which is associated with telomere erosion, oncogene-induced and stress-induced premature senescence (SIPS) occurring in response to different stress stimuli^3^. Cancer cells, due to their ability to overcome the effect of telomere shortening, evade replicative senescence but can undergo SIPS^4^. A number of studies showed development of the senescence phenotype of cancer cells as the outcome of chemotherapy in vitro and in vivo^5,6^. Moreover, induction of SIPS requires lower doses of chemotherapeutics than those required to kill cancer cells^7^. However, there is some evidence proving that senescence of cancer cells is transient and might lead to cancer relapse^8–12^.

Autophagy is a well-known evolutionarily conserved catabolic program for the degradation of proteins and other subcellular elements through lysosomal lysis. Autophagy serves as a prosurvival mechanism that adapts cells to stress conditions^13,14^, but may also lead to cell demise called programmed cell death type II^15^, which is distinct from apoptosis and other cell death modes^16,17^.

It has been shown that in normal fibroblasts autophagy is activated upon induction of senescence and contributes to the establishment of senescence^18^. However, the connection between autophagy and senescence in normal and cancer cells seems to be much more complex^19–21^.

A characteristic feature of macroautophagy (herein referred to as autophagy) is the formation of autophagosomes, which fuse with lysosomes, wherein their cargo is degraded^22^. Elevated basal autophagy, characteristic for a variety of tumors, has become critical for their metabolism^23^. There are plethora of reports demonstrating that autophagy inhibition leads to increased efficiency of pharmacological anticancer treatment and to increased effectiveness of radiotherapy^24,25^. At present, the most promising approach seems to be a combined anticancer therapy, in which autophagy is induced and simultaneously blocked at the degradation stage^26,27^.

In this study, we present a new compound, tacrine-melatonin heterodimer C10, synthesized by us as an acetylcholinesterase (AChE) and butyrylcholinesterase (BuChE) inhibitor and potential anti-Alzheimer’s drug^28^, which possesses antiproliferative properties due to autophagy modulation. Heterodimer C10 simultaneously induces autophagy and blocks it at the degradation stage. These properties of C10 place this compound among promising anticancer agents.

## Results

### C10 has cytostatic/cytotoxic effect on MCF-7 cells

**Fig. 1:**
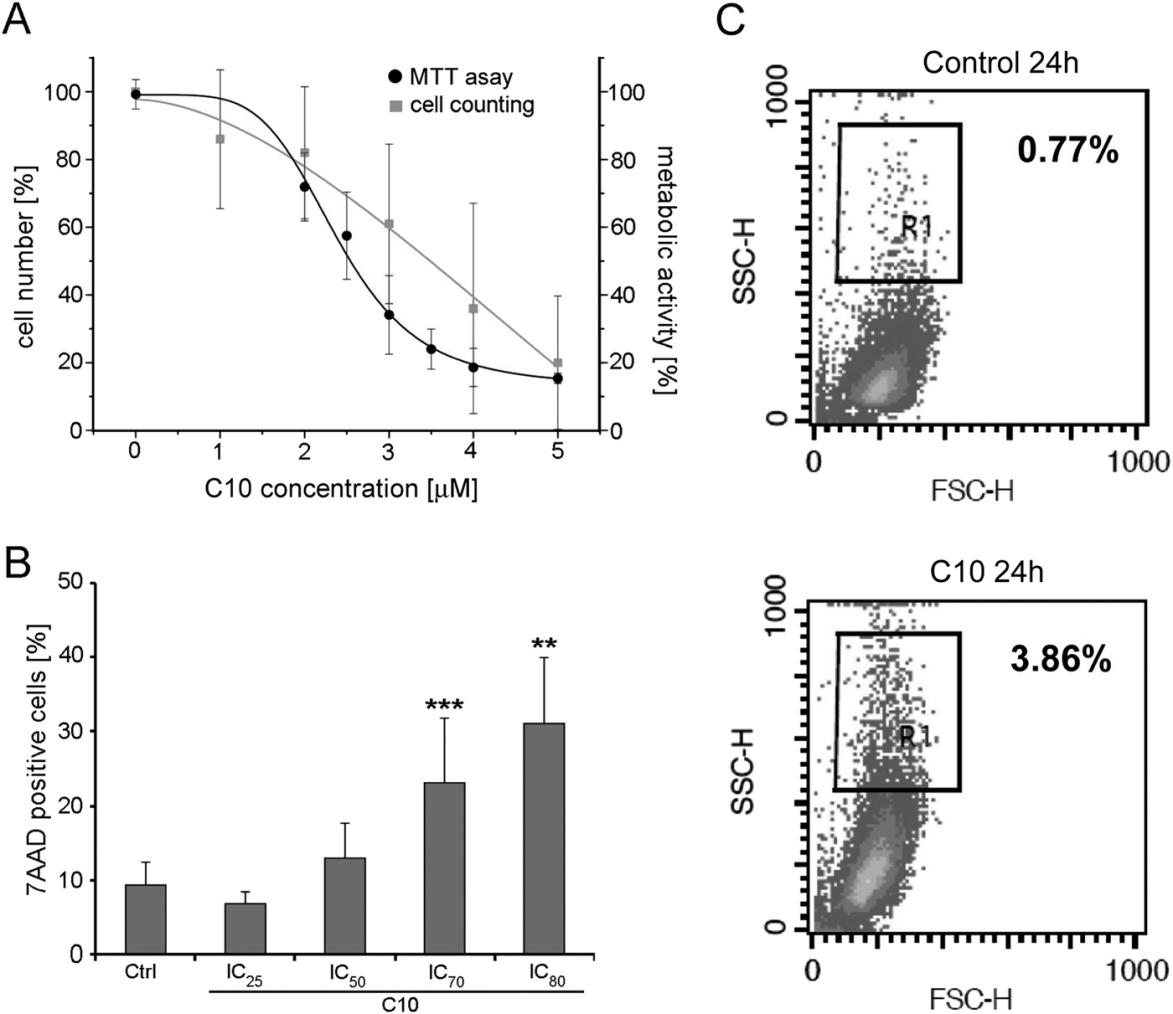
C10 has cytostatic/cytotoxic effect on breast cancer MCF-7 cells. **a** Viability of MCF-7 cells treated for 24 h with indicated concentrations of C10 measured by MTT assay (4 independent experiments) and cell counting with trypan blue exclusion (8 independent experiments). The data were calculated as the percentage of the control. **b** Analysis of cell death by 7AAD assay after treatment for 24 h with indicated inhibitory concentrations (IC) of C10 (13 independent experiments). The data were calculated as the percentage of total cell population. **P < 0.01 and ***P < 0.001 vs. control. **c** Representative dot plots of granularity analysis by flow cytometry for control cells and cells treated with IC_50_ of C10 for 24 h (one of 3 independent experiments)

**Fig. 2:**
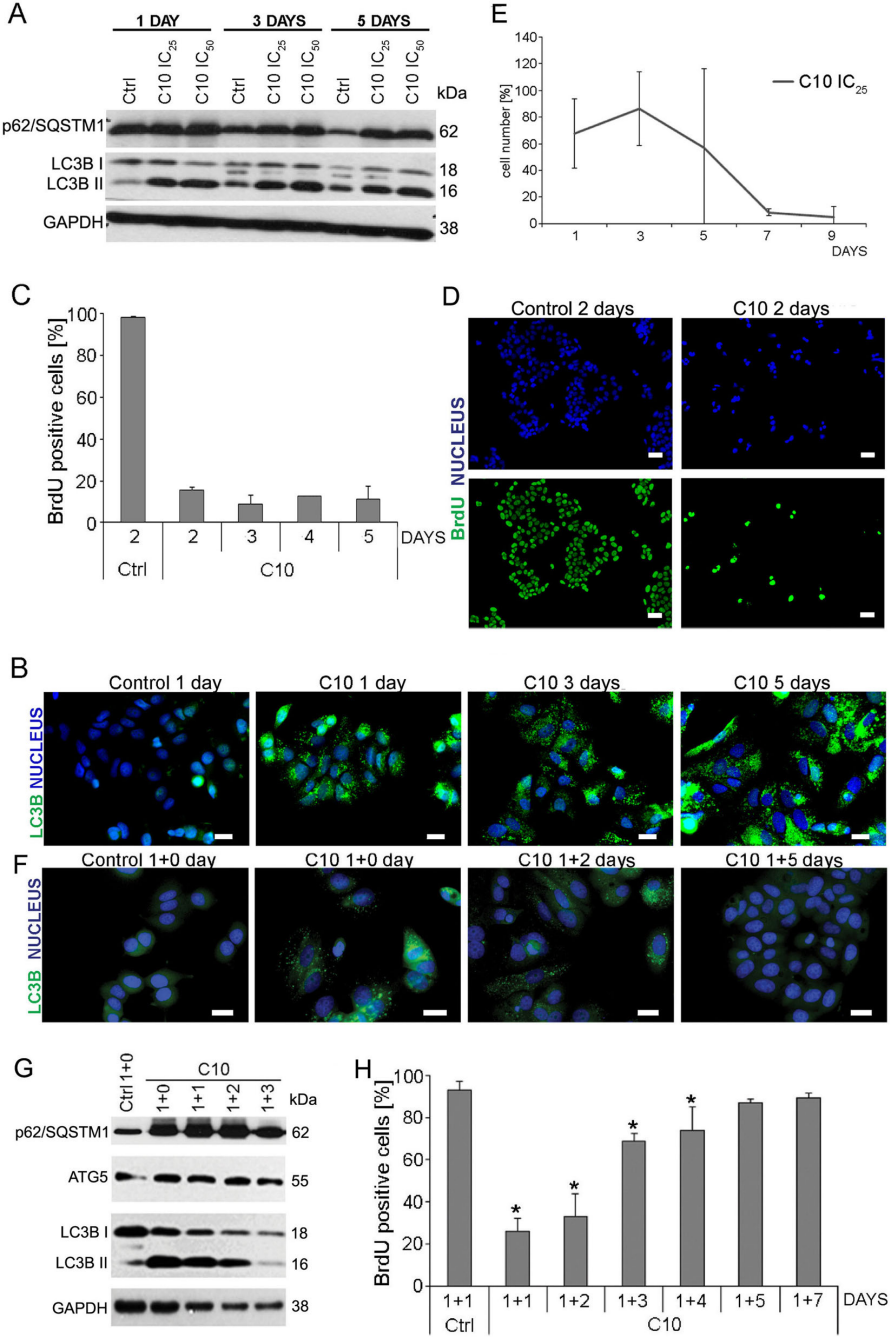
Cell fate after prolonged and transient treatment with C10. **a**–**c** MCF-7 cells were treated with IC_25_ (2 µM) or IC_50_ (3 µM) dose of C10 for the indicate time. **a** Representative western blots of p62/SQSTM1 and LC3B protein level. **b** Representative immunofluorescence images of cells stained for LC3B (green) and nuclei with DAPI (blue) (one of 2 independent experiments). Scale bars: 20 µm. **c** Quantitative analysis of BrdU incorporation in MCF cells and (**d**) representative immunofluorescence images of BrdU-positive cells (green) and nuclei stained with DAPI (blue) (3 independent experiments). Scale bars: 50 μm. The data were calculated as the percentage of total cell population. **e** The viability measured by cell counting with trypan blue exclusion. The data were calculated as the percentage of the control. **f**–**h** MCF-7 cells were treated with IC_50_ dose of C10 for 1 + n days. **f** Representative immunofluorescence images of cells stained for LC3B (green) and nuclei with DAPI (blue) (one of 8 independent experiments). Scale bars: 20 µm. **g** Representative western blots of ATG5, p62/SQSTM1 and LC3B protein level. **h** Quantitative analysis of BrdU incorporation in MCF cells (4 independent experiments). Scale bars: 50 μm. The data were calculated as the percentage of total cell population. *P < 0.05 vs. control

C10 is a compound containing a tacrine and melatonin part, linked by a ten carbon chain (Supplemental Fig. 1A), synthesized according to the procedure described previously^28^. We show that, 24 h after treatment with C10, the number of MCF-7 cells and their metabolic activity (measured by MTT) decreased in a dose-dependent manner (Fig. 1a). The IC_50_ dose of C10 was calculated from MTT and cell counting curves to be in the range of 2.5–4 μM depending on the batch. The cell death rate after treatment with IC_50_ of C10 (measured by 7AAD) was close to the level for untreated cells. Thus, the treatment with IC_50_ dose of C10 for 24 h has cytostatic effect, however, higher doses of C10 caused cell death after 24 h treatment (Fig. 1b). Moreover, prolonged treatment with IC_50_ concentration led to cell death at the third day. Similar results were obtained after treatment with IC_25_ dose of C10; however, cells died at fifth day (Fig. 2E). Altogether, C10 has cytostatic effect on cells but prolonged treatment with this compound is cytotoxic and results in death after few days. Interestingly, components of the heterodimer, tacrine and melatonin, applied together in concentrations equal to IC_70_ of C10 did not affect the death rate of MCF-7 cells (measured by MTT and 7AAD assays) (Supplemental Fig. 1B). Additionally, in a dose-dependent manner treatment with melatonin provoked only a slight decrease in cell metabolic activity, while tacrine evoked a pronounced decrease, starting from 50 μM concentration. On the other hand, C10 caused a 50% drop at the concentration of 3–5.5 μM, depending on cell type (Supplemental Fig. 1D).

### C10 causes increase in the number and size of autophagic vesicles in MCF-7 cells

Analysis of cell morphology revealed that a 24 h treatment with IC_50_ dose of C10 led to an increase in cellular granularity (Fig. 1c), which could suggest modulation of the autophagic process^29^.

**Fig. 3:**
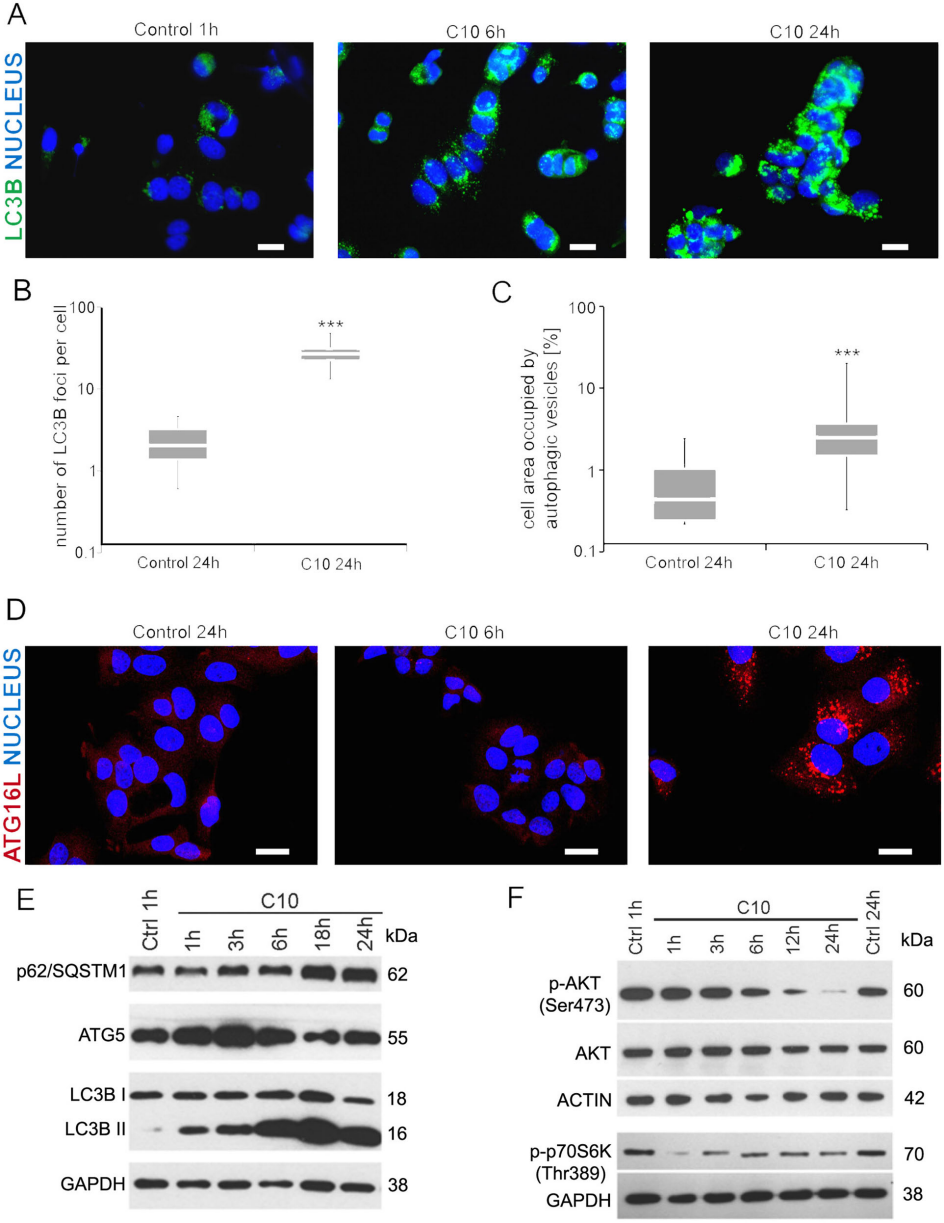
C10 induces autophagy in MCF-7 cells. MCF-7 cells were treated with IC_50_ dose of C10 or vehicle for indicated time. **a, d** Representative immunofluorescence images of cells stained for LC3B (green) in (**a**) or ATG16L (red) in (**d**); nuclei stained with DAPI (blue) (one of 2 or 3 independent experiments for ATG16L and LC3B, respectively). Scale bars: 20 µm. **b** Quantification of LC3B foci per cell performed using immunofluorescence microscopy. Foci were counted in at least 350 cells in each of 3 independent experiments. The data represent median and IQR. ***P < 0.001 vs. control. **c** Quantitative analysis of cell area occupied by autophagic vesicles in control cells and cells treated with IC_50_ dose of C10 based on electron microscopy images. The surface of vesicles in at least 30 cells per sample was counted. The data represent median and IQR. ***P < 0.001 vs. control. **e**, **f** Representative western blots showing protein level of (**e**) p62/SQSTM1, ATG5, LC3B and (**f**) phospho-AKT (p-AKT), AKT, phospho-p70S6K (p-p70S6K)

ATG proteins as well as autophagy adaptor protein p62/SQSTM1 and microtubule-associated protein 1 light chain 3 (LC3) are involved in cargo sequestration during autophagosome formation. The most commonly used parameter, which is a sign of autophagy modulation, is lipidation of LC3, which is manifested by the appearance of LC3 signal in punctate structures^30^. Indeed, we observed that already after 6 h of C10 treatment, MCF-7 cells exhibited increased number of LC3B puncta (Fig. 3a). Quantitative analysis revealed that, after 24 h, the number of LC3B foci per cell increased 7.5 times in comparison with control cells (Fig. 3b). Moreover, electron microscopy analysis showed a C10-induced rise in cell area occupied by autophagic vesicles, which was due to an increase in their number and size (Fig. 3c). Increased number of autophagic vesicles may be a result of autophagy induction or/and its blockade^31^.

### C10 induces autophagy in MCF-7 cells

Western blot analysis showed an increase in the ATG5 level that peaked already after 3 h of treatment with C10 and returned to the basal level after 24 h (Fig. 3e). This transient increase in ATG5 level provides evidence of autophagy induction.

It was demonstrated that the ATG5–ATG12–ATG16L complex is required for the elongation of the isolation membrane and is present on the phagophore but not on a mature autophagosome^32,33^. Thus, increase in ATG16L-positive puncta is widely used as a marker of autophagy induction. We observed that C10 evoked a detectable increase in ATG16L-positive puncta in MCF-7 cells after 24 h of treatment (Fig. 3d). Subsequently, we found that the level of LC3B II protein augmented progressively within 24 h of treatment (Fig. 3e).

As melatonin has been shown to induce autophagy in different types of tumours^34,35^, we examined the effect of C10 components on autophagy markers. Treatment with tacrine or melatonin in a dose equal to IC_50_ for C10 did not cause any pronounced change in LC3B II, ATG5 or p62/SQSTM1 protein levels (Supplemental Fig. 1C). Similarly, treatment with 1 mM melatonin did not affect the number of LC3B foci in MCF-7 cells (Supplemental Fig. 1E). This suggests that the tacrine-melatonin hybrid possesses different properties than its individual components.

It is well known that AKT/mTOR signalling pathway is involved in autophagy regulation^36^. Indeed, time-dependent analysis of the active, phosphorylated AKT (p-AKT) protein level showed its progressive decrease that became evident after 6 h of treatment with C10 and a drop to a hardly detectable level after 24 h. The attenuation of p-AKT was preceded by a decrease in the level of phosphorylated p70S6 kinase (p-p70S6K), the mTOR substrate (Fig. 3f). These results suggest that in MCF-7 cells C10 evokes inhibition of mTOR activity followed by a decrease in p-AKT kinase level.

### C10 blocks autophagic flux

**Fig. 4:**
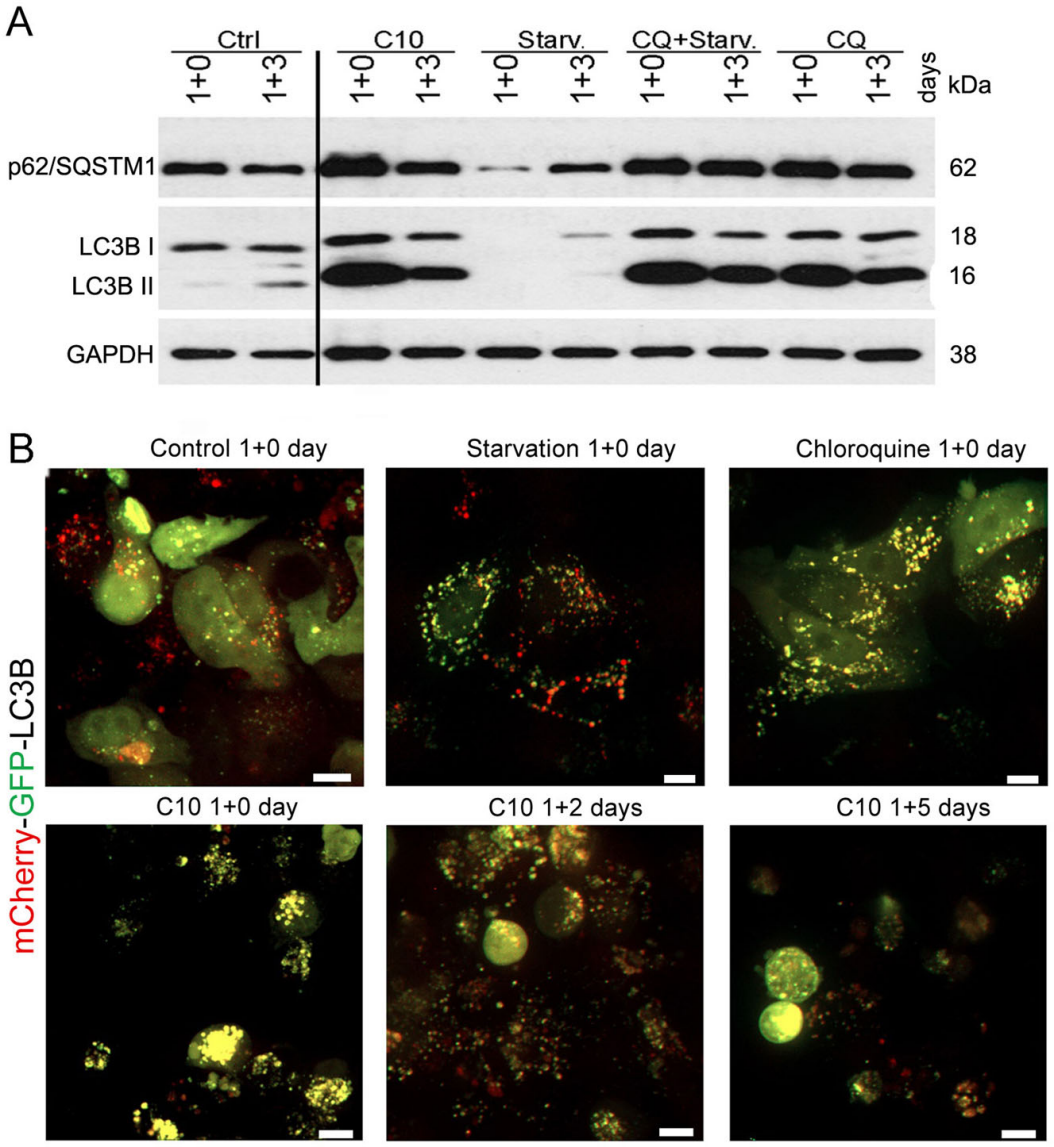
C10 blocks autophagic flux in MCF-7 cells. **a** Representative western blot of LC3B and p62/SQSTM1 protein level in MCF-7 cells cultured in full or serum depleted medium or medium containing IC_50_ dose of C10 or 10 µM chloroquine for 24 h (1 day) followed by n days of culture in drug free full medium (1 + n). **b** Representative confocal images showing colocalization of mCherry and GFP signals in cells transiently transfected with plasmid coding for mCherry-GFP-LC3B and treated as described above (one of 4 independent experiments). Scale bars: 10 µm. CQ chloroquine, Starv starvation

To better understand the impact of C10 on autophagy, we analysed the autophagic flux during C10-treatment in comparison with starvation-induced autophagy and CQ-induced blockade of autophagy. While cell starvation for a few hours leads to autophagy induction and increase in autophagy markers, it may cause disappearance of autophagy proteins if prolonged^37^. Accordingly, 1-day starvation of MCF-7 cells resulted in a complete disappearance of autophagy markers. In contrast, CQ and C10 caused accumulation of autophagy proteins (p62/SQSTM1, LC3B II) with time (Figs. 3e and 4a). This data suggests that apart from stimulating autophagosome formation, C10 also blocks the autophagic flux at the late stage.

To confirm this assumption, we monitored the autophagic flux with a mCherry-GFP-LC3B construct. GFP fluorescence is quenched after fusion of an autophagosome with an acidic lysosome, while mCherry fluorescence is not affected by acidic environment. Thus, autolysosomes can be visualized as red LC3B foci. On the other hand, when the autophagosome-lysosome fusion is blocked or when autolysosomal pH is increased, both mCherry and GFP can be detected resulting in yellow LC3B puncta^31,38^ In control cells, we found both red and yellow LC3B puncta as well as dispersed yellow fluorescence in the cytoplasm, which indicates the presence of a non-lipidated LC3B I form. As anticipated, starvation led to an increase in the number of LC3B puncta with predominance of red ones, indicating that the autophagic flux is enhanced. In contrast, CQ treatment evoked accumulation of mostly yellow puncta, which was the consequence of autolysosome maturation blockade. Treatment with C10 yielded a similar pattern to that observed for CQ (Fig. 4b). Overall, these results indicate that C10 inhibits the autophagic flux at the late stage.

### C10 affects maturation/degradation of autolysosomes

**Fig. 5:**
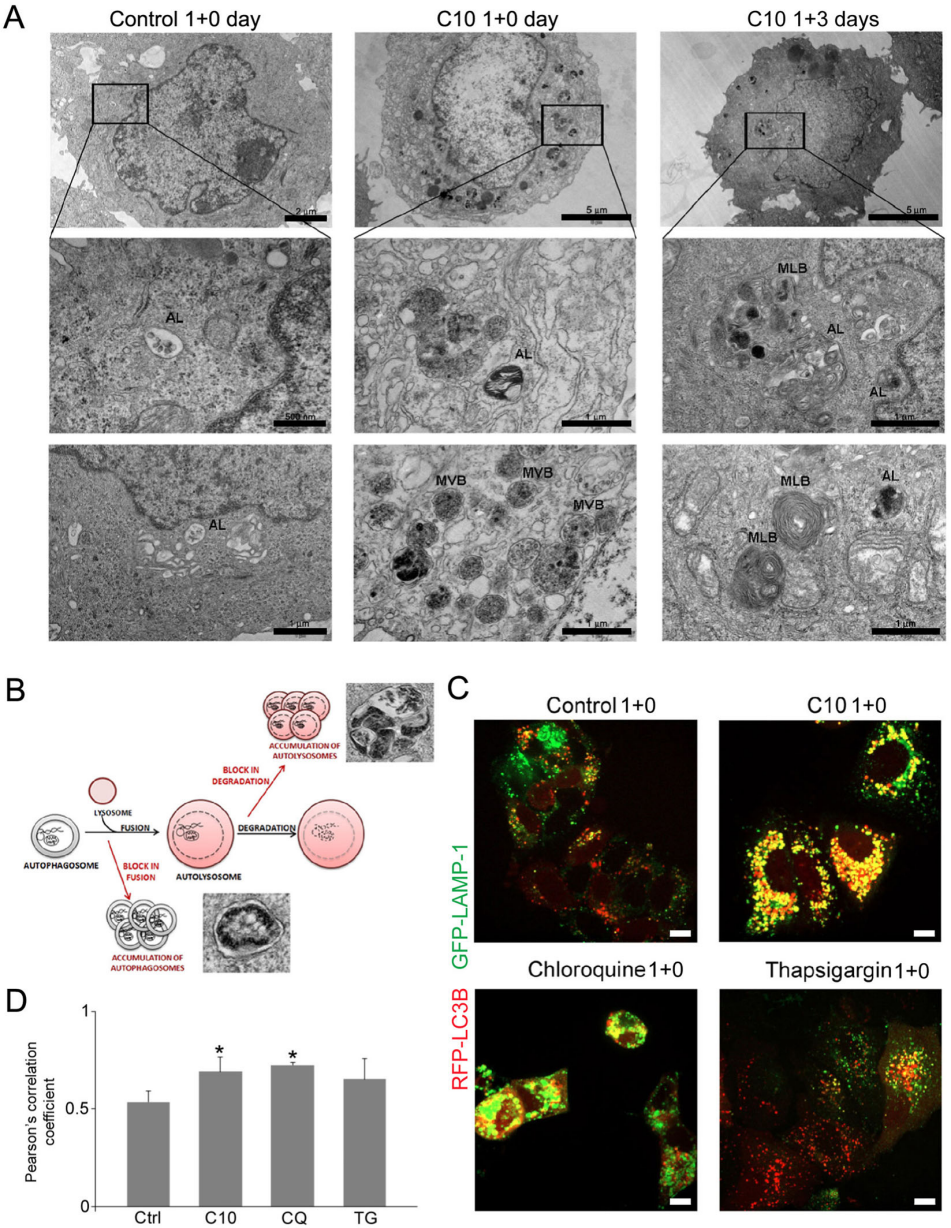
C10 affects autolysosomal degradation in MCF-7 cells. **a** Electron microscopy images of cross-sections of cells treated with IC_50_ dose of C10 or vehicle for 1 + n days. The images show typical cells on the subsequent days following treatment (upper panel) and their magnified parts with representative autophagic vesicles (lower panels). AL-autolysosome, MVB-multivesicular body, MLB-multilamellar body. **b** Scheme illustrating possible ways of autophagy blockade at the late stage with representative electron microscopy images of autophagic vesicles. **c**, **d** MCF-7 cells were transiently cotransfected with mRFP-LC3B and GFP-LAMP-1 plasmids and cultured in full medium with or without IC_50_ dose of C10 for 24 h (1 day). Cells treated with 10 µM chloroquine or 5 µM thapsigargin were used as positive and negative controls, respectively. **c** Representative confocal images of LC3B (red) and LAMP-1 (green) proteins localization (one of 5 independent experiments). Scale bars: 10 µm. **d** Pearsons’ correlation coefficients for colocalization of LC3B with LAMP-1 (4 independent experiments). CQ-chloroquine, TG-thapsigargin. *P < 0.05 vs. control

Blockade of the autophagic flux may be a result of two processes: inhibition of autophagosome and lysosome fusion and/or impairment of lysosomal degradation. Inhibition of fusion leads to accumulation of autophagosomes. However, attenuation of lysosomal function precludes degradation of autolysosome cargo, which results in an increase in the number of non-degraded autolysosomes^39^. Autophagosomes are double-membrane vesicles, but their fusion with lysosomes leads to formation of single-membrane autolysosomes the electron microscopy images of which are presented on the scheme (Fig. 5b). We showed by electron microscopy that a 24 h-treatment of MCF-7 cells with C10 caused accumulation of big vesicles with dense material (Fig. 5a). The vesicles had single membrane and were bigger than those observed in control cells. Moreover, in C10-treated cells, we observed vesicles similar to multivesicular bodies (MVBs).

Accumulation of single-membrane vesicles suggests that C10 inhibits the autophagic flux through suppression of autolysosome degradation. To test this assumption we utilized mRFP-LC3B and GFP-LAMP-1 plasmids and examined colocalization of LC3B puncta (autophagosomal marker) with LAMP-1 (lysosomal marker) in C10-treated MCF-7 cells. We observed high colocalization of the two signals after C10 treatment, similarly as in cells treated with CQ but not in control or thapsigargin-treated cells (Fig. 5c). Thapsigargin (TG), by raising cytosolic calcium concentration, inhibits vesicle fusion^40^. Quantitative analysis of LC3B and LAMP-1 proteins colocalization demonstrated significantly higher Pearsons’ correlation coefficient for C10- and CQ-treated cells vs. control and TG-treated cells (Fig. 5d).

Altogether this data reveals that C10 inhibits the autophagic flux in MCF-7 cells, which leads to accumulation of single-membrane autolysosomes with non-degraded cargo.

### Prolonged treatment with C10 leads to massive cell death

To examine the effect of prolonged C10-treatment, MCF-7 cells were grown in the presence of IC_25_ or IC_50_ dose of C10 for several days (up to 9 days). This led to accumulation of autophagy markers such as LC3B II and p62/SQSTM1 at the protein level and LC3 foci that appeared within 24 h and remained visible during the following days (Fig. 2a,b). Additionally, a decrease in the number of BrdU-positive cells (to 20%) was observed during the treatment with heterodimer (Fig. 2c,d). This suggests that prolonged treatment with C10-evoked autophagy blockade and inhibition of proliferation. Interestingly, cell counting showed that the cell number remained nearly unchanged within 4 days and then dramatically dropped down indicating rapid and massive cell death on the 5th or 6th day. (The high standard deviation reflects differences in the time of cell death observed in different experiments) (Fig. 2e).

### Transient treatment with C10 leads to cellular senescence or regrowth

To reveal cell fate after autophagy modulation evoked by transient treatment of MCF-7 cells with C10 we analysed some autophagy markers after C10 removal. We observed progressive disappearance of LC3B-positive foci and a decrease in the level of proteins such as p62/SQSTM1 and LC3B II (Fig. 2f,g). Similar effect could be observed after withdrawal of CQ but not after discontinuation of starvation (Fig. 4a). However, after C10 withdrawal, the ATG5 protein level was similar to this observed in the control (Fig. 2g). Autophagic flux analysis using mCherry-GFP-LC3B plasmid disclosed progressive appearance of red vesicles suggesting autophagy resumption after C10 washout (Fig. 4b). Moreover, electron microscopy revealed formation of empty-like vesicles with degraded cargo and multilamellar bodies (MLBs) on the 3rd day of culture without the drug (Fig. 5a). Formation of MLBs is associated with degradation of lipid-rich autophagic vesicles such as autophagosomes with numerous intraluminal vesicles or MVBs^41^.

In contrast to prolonged treatment that had an antiproliferative effect on MCF-7 cells followed by cell death, drug removal after 24 h treatment with IC_50_ dose of C10 caused only transient inhibition of cell proliferation. On day 1+1 (1st day after C10 removal) we found 30% of BrdU-positive cells and then their number increased with time to the control level on the 5th day of culture without C10 (Fig. 2h).

**Fig. 6:**
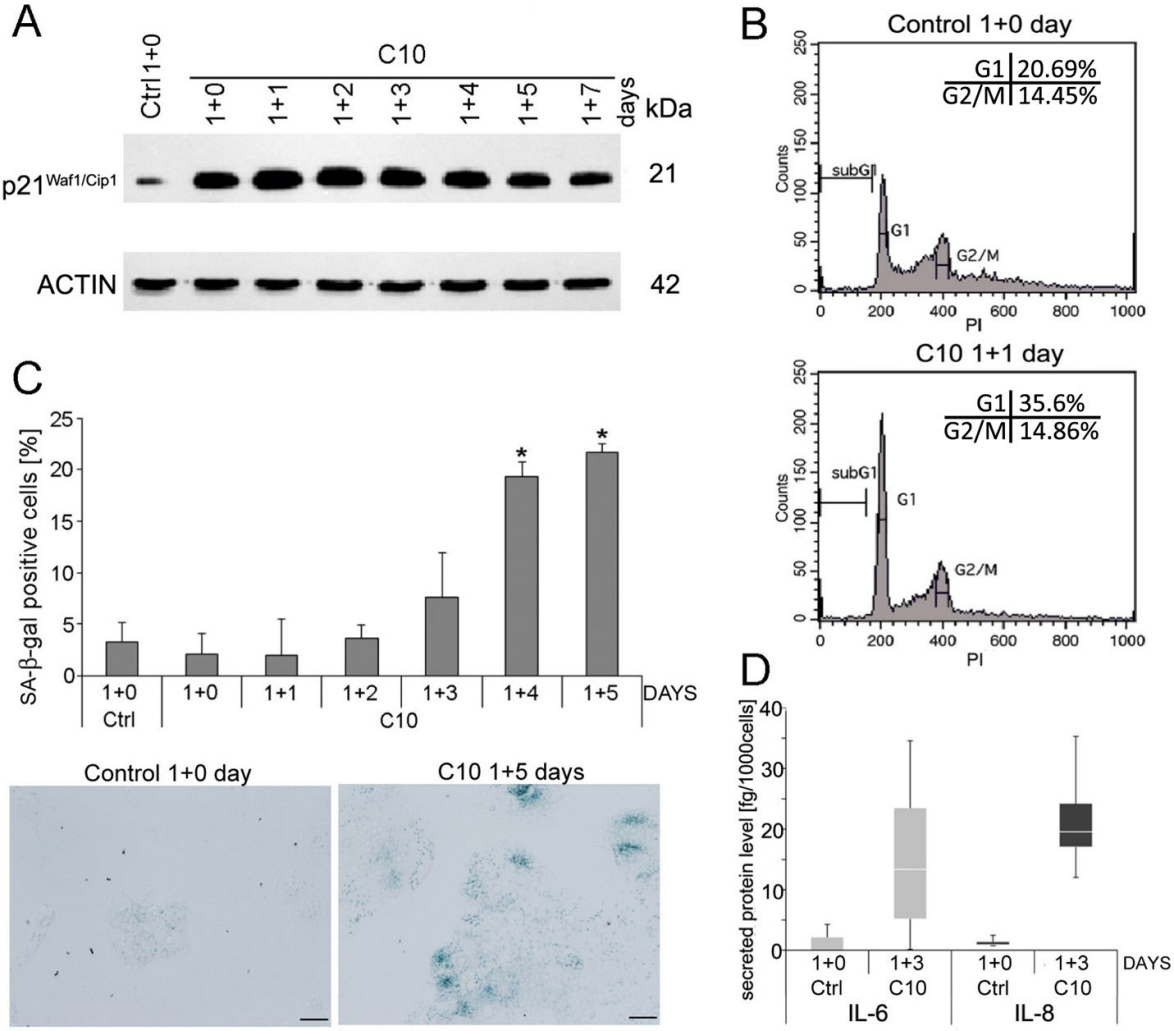
Transient treatment of C10 induces senescence of MCF-7 cells. MCF-7 cells were treated with IC_50_ dose of C10, and subjected to analysis of cellular senescence markers on the 1 + n day of treatment. **a** Western blot analysis of p21^WAF1/CIP1^ protein level. **b** Representative histograms of cell cycle analysis by flow cytometry (one of 3 independent experiments). **c** Quantitative analysis from 4 independent experiments of SA-β-galactosidase positive cells and representative phase contrast images (blue staining represents SA-β-galactosidase activity). Scale bars: 20 µm. The data were calculated as the percentage of total cell population. *P < 0.05 vs. control. **d** Quantitative analysis of 4 independent experiments of IL-6 and IL-8 level estimated by flow cytometry. Data represents median and IQR

Cellular senescence is associated with cell cycle arrest and may occur in the cancer cells in response to stress such as treatment with chemotherapeutic drugs^8,42^. Therefore, we have checked senescence markers upon transient C10-treatment. We observed a cell cycle arrest in the G1 phase and an increased level of the cell cycle inhibitor, p21^Waf1/Cip1^ (Fig. 6a,b). C10 removal resulted in an increased number of SA-β-gal-positive cells that amounted to 20% on the 4th day (Fig. 6c). Moreover, elevation of secreted proinflammatory cytokines, such as interleukin 6 (IL-6) and 8 (IL-8), characteristic for senescent cancer cells^43^, was observed (Fig. 6d).

### C10 enhances autophagy and blocks autophagic flux in HCT 116 cells and normal fibroblasts

**Fig. 7:**
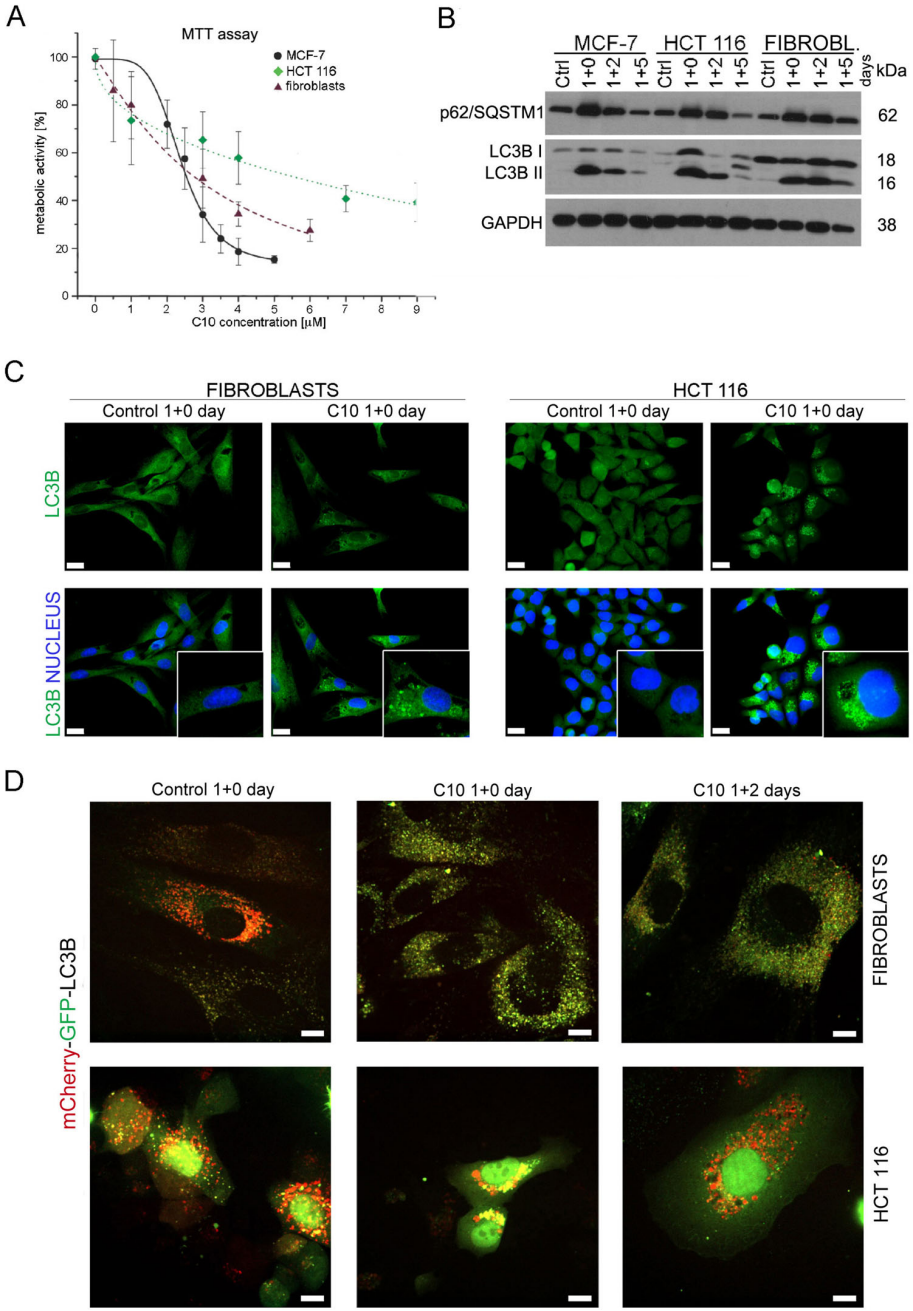
C10-evoked autophagy enhancement and blockade in HCT 116 cancer cells and normal fibroblasts. **a** The viability of MCF-7 cells, HCT 116 cells and normal human fibroblasts treated for 24 h (1 day) with indicated concentrations of C10 measured by MTT assay (4 independent experiments). The data were calculated as the percentage of control. **b**–**d** HCT 116 cells, normal human fibroblasts or MCF-7 cells were treated with appropriate IC_50_ dose of C10 for 1 + n days. **b** Representative western blot analysis of p62/SQSTM1 and LC3B protein level. (**c**) Representative immunofluorescence images of LC3B (green) and nuclei stained with DAPI (blue) (one of 3 independent experiments). Scale bars: 20 µm. **d** Cells were transiently transfected with mCherry-GFP-LC3B plasmid. Representative confocal images of mCherry and GFP signal colocalization (one of 4 independent experiments). Scale bars: 10 µm

To verify whether C10 affects autophagy in other cell types we used HCT 116 colon cancer cells and normal human fibroblasts treated for 24 h (1 day) with IC_50_ doses of C10 - 5 μM and 3.5 μM, respectively. The MTT assay showed that HCT 116 cells and fibroblasts were less sensitive to C10 than MCF-7 cells (Fig. 7a). Nonetheless, C10 enhanced autophagy in all types of tested cells, which was confirmed by an increase in LC3B II protein level and in the number of LC3B-positive vesicles (Fig. 7b,c).

These changes were accompanied by accumulation of p62/SQSTM1 protein that suggested blockade of autophagic flux. To verify it we analysed the autophagic flux by transfecting cells with mCherry-GFP-LC3B plasmid. In untreated fibroblasts and HCT 116 cells red and yellow LC3B puncta as well as green and red fluorescence (LC3B I form) dispersed in the cytoplasm were visible. One-day C10-treatment evoked a blockade of the autophagic flux confirmed by accumulation of yellow foci in fibroblasts and a less pronounced effect in HCT 116 cells (Fig. 7d). That blockade was relieved within 2 days after drug removal when the red foci appeared, and p62/SQSTM1 and LC3B II proteins level decreased (Fig. 7b).

Altogether, we have demonstrated that the effect of C10 on autophagy is not limited to MCF-7 cells but occurs also in other cancer (HCT 116) and normal (human fibroblasts) cells, however with different intensity.

## Discussion

We demonstrated that tacrine-melatonin heterodimer C10, originally synthesized as an anti-Alzheimer’s drug with inhibitory effect on human erythrocyte AChE, has anticancer properties. The effect of C10 on MCF-7 cells was not caused by AChE inhibition since the compound did not significantly change AChE level and activity in these cells (Supplemental Figs. 2A, B). We observed that the anticancer properties of C10 were not due to the properties of melatonin or tacrine either but resulted from the particular hybrid structure of C10. We have found that C10 induces cellular senescence of MCF-7 cells which, most importantly, is preceded by modulation of the process of autophagy. In C10-treated MCF-7 cells we observed typical markers of autophagy initiation, such as rapid autophagic vesicle formation, confirmed by an increase in ATG16L-positive puncta, and transient elevation of ATG5 protein level followed by an increase in LC3B II protein level. Such changes were not observed when cells were treated with melatonin or tacrine alone at the concentration equal to IC_50_ dose of C10. Also, the PI3K/AKT/mTOR signalling pathway, one of the key pathways involved in autophagy regulation^30^, seemed to be affected by C10. C10 affected activation of p70S6K, a mTORC1 substrate, followed by a decline in p-AKT protein level without affecting total mTOR protein level. Attenuation of mTORC1 activity, enhanced by a decrease in p-AKT, together with elevated number of ATG16L-positive vesicles argue for autophagy induction.

It is believed that, once initiated, the process of autophagy proceeds until complete degradation of cargo and disappearance of autolysosomes^30^. In the case of C10, the increase in autophagy initiation markers was accompanied by accumulation of autolysosomes with undegraded cargo. This indicates that autophagy was induced but, simultaneously, the autophagic flux was inhibited as a result of a block in autolysosome degradation. Undegraded autolysosomal cargo counteracts mTORC1 reactivation that is required for autophagic lysosomal reformation (ALR)^44^. In this process nascent lysosomes are formed from autolysosomes, which facilitates autophagy completion. On the other hand, enhanced formation of autophagosomes during autophagy induction requires functional lysosomes for cargo degradation, the number of which could be insufficient because of unsettled ALR. Due to its dual action on autophagy C10 represents a novel autophagy modulator.

Most of known compounds that affect autophagy are either inducers or inhibitors of this process. Autophagy inhibitors, CQ and its derivatives, are tested in clinical trials^2^. However, drugs that are able to affect autophagy in a dual mode, by inducing and inhibiting the process, seem to represent a new strategy in anticancer therapy. Recently, it has been shown that a naphthalene sulphonamide derivative (ML-9) has such a dual effect on autophagy. This drug stimulates autophagosome formation by downregulating the AKT/mTOR pathway and inhibits the autophagic flux by increasing lysosomal pH and affecting autolysosomal maturation in LNCaP prostate carcinoma cells^45^. Recently it has also been demonstrated that N6-isopentenyladenosine is able to induce and inhibit autophagy by targeting AMPK and Rab7, which leads to AMPK-dependent apoptosis of melanoma cells^46^. Another compound, Autophagy Modulator with Dual Effect (AMDE-1), had a similar effect on autophagy in some cell lines such as MEFs, A549, Sasos2 and HEK293. AMDE-1 action consists of AMPK-mediated autophagy induction and its blockade by inhibiting the activity of lysosomal cathepsin B without changing lysosomal acidity^27^. We cannot exclude that C10, as a weak base with lipophilic properties, may be preferentially sequestered in acidic cellular compartments such as lysosomes or autolysosomes. This may lead to attenuation of their degradation capacity and, in consequence, to autophagy perturbations.

Moreover, it has been shown that autophagy inhibitors with lysosomotropic or ionophore properties are able to parallelly activate a noncanonical autophagy pathway that drives LC3 lipidation on endolysosomal membranes^47^. In the case of C10, attenuation of mTORC1 activity 1–3 h after treatment proves the induction of canonical autophagy. However, inihbition of autophagy by C10 preceded the observed increase in the number of ATG16L foci. C10-evoked autophagy blockade leads to accumulation of autolysosomes with undegraded cargo and may cause rupture of these vesicles. Therefore, we cannot exclude that these damaged autophagic vesicles may, in consequence, induce noncanonical autophagy.

Enhanced autophagy process, when incomplete (due to co-treatment with autophagy inducer and inhibitor), leads to cell degeneration followed by cell death^26^. C10-induced MCF-7 cell death was observed for doses higher than the IC_50_ dose. Moreover, long-term treatment of MCF-7 cells with the IC_50_ or IC_25_ dose of C10 led to persistent impairment of autophagy and inhibition of cell proliferation until the 5th or 6th day when massive cell death was observed. Autophagy modulation by C10 and absence of functional caspase 3 in MCF-7 cells argue for autophagy-dependent and caspase-independent cell death. Similarly, prolonged treatment with C10 of HCT 116 cells, in which all caspases are functional, evoked autophagy blockade followed by massive cell death on the 6th day (data not shown). This suggests that C10 is able to induce autophagy-dependent cell death in various cancer cells.

In contrast to cell death observed after prolonged C10-treatment relievement of the autophagy blockade led to the regrowth of cells. However, cells that were unable to resume proliferation after stress caused by autophagy perturbation underwent senescence (20%). One can argue that C10 may change pH and lower the activity of SA-β-gal as it was shown in the case of CQ^48^. This cannot be excluded but cell proliferation can still be observed after C10 removal proving that at least not all cells undergo senescence. Another scenario is also possible, namely reversibility of cancer cell senescence^8,43,49,50^. However, MCF-7 cells, may undergo both irreversible or reversible cell cycle arrest at least after various treatments with doxorubicin^49,51^.

There are some reports that autophagy inhibition induces cell death in cancer cells resistant to common therapies; moreover, in some cases, it may restore anticancer drug sensitivity^26^. It was demonstrated that autophagy was elevated after radiotherapy but CQ applied after irradiation inhibited the post-irradiation recovery process^25^. It was also found that autophagy inhibition and chemotherapeutics can give synergistic effects in different cancer models^52,53^. On the other hand there is also evidence that, in some cases, autophagy inducers may enhance anticancer therapy^54,55^. In this context compounds that are able both to induce and inhibit autophagy may cause extreme metabolic energy exhaustion that leads to cell stress and death. Moreover, accumulated vesicles with undegraded cargo that cannot be recycled may further contribute to cell stress.

Summing up, we showed that C10 causes autophagy modulation in lower doses than other autophagy dual mode agents, namely ML-9 and AMDE-1^27,45^, and exerts higher cytotoxicity than CQ on MCF-7 breast cancer cells. Due to C10 action on both the initiation and degradation stage of autophagy it seems to be a more potent anticancer agent than inhibitors presently used in therapy, such as CQ derivatives. C10 acts on a universal target i.e., the autophagy process, which is involved in tumorigenesis and drug resistance. Moreover, inefficient functioning of autophagy may induce cell death even in cancer cells resistant to caspase-dependent apoptosis. Currently, we are dissecting this possibility by using the protocol of prolonged cancer cell treatment with C10. Moreover, we can speculate that due to the frequently observed higher basal level of autophagy in different types of cancer cells and their higher nutrient demand^23,26^, such autophagy modulation as evoked by C10 may be more debilitating for cancer than for normal cells.

## Materials and Methods

### Reagents and antibodies

The following antibodies were obtained from commercial sources: rabbit anti-AChE (1:500, STI, bs-2511R), mouse anti-p21 (1:500, Sigma-Aldrich, P1484), mouse anti-p62 (1:1000, BD Biosciences, 610832), rabbit anti-ATG5 (1:200, Cell Signaling, #2630), mouse anti-ATG16L (1:500, MBL, M150-3), rabbit anti-LC3B (1:500, Sigma-Aldrich, L7543), rabbit anti-AKT (phospho Ser473) (1:1000, Cell Signaling, #4060S), rabbit anti-AKT (1:1000, Cell Signaling, #4691S), rabbit anti-p70S6K (phospho Thr389) (1:500, Cell Signaling, #9208P) and mouse anti-β-ACTIN (1:50,000, Sigma-Aldrich, A1978) or mouse anti-GAPDH (1:50,000, Milipore, MAB374), anti-rabbit Alexa 488 and anti-mouse Alexa 555 (1:500, Life Technologies, A11008 and A31570) secondary antibodies. Chloroquine was purchased from Lab Empire (CHL919), Bafilomycin A and Thapsigargin from Sigma-Aldrich (B1793 and T9033).

### Cell culture and treatment

MCF-7 cells and human primary dermal fibroblasts were obtained from ATCC. The human colon HCT 116 cancer cell line was kindly provided by Dr. Bert Vogelstein (Johns Hopkins University, Baltimore, MD). Authentication of MCF-7 and HCT 116 cell lines was performed by Cell Line Authentication IdentiCell using STR profiling in 2016.

MCF-7 cells and fibroblasts were cultured in DMEM medium (Sigma-Aldrich, D5546), HCT 116 in McCoy’s medium (Lonza, BE12_168F). Media were supplemented with 10% fetal bovine serum (FBS) (Cytogen, S181H), 2mM L-glutamine and Antibiotics Antimycotic solution (Sigma-Aldrich, G7513 and A5955). Cells were kept in humidified atmosphere (37 °C and 5% CO_2_ in the air). The cells were seeded 24 h before treatment at a density of 6 × 10^3^ cells/cm^2^ (MCF-7) or 5 × 10^3^ cells/cm^2^ (HCT 116 and fibroblasts).

To check long-term effects of heterodimer C10, cells were treated with IC_50_ dose of C10 for 24 h and then cultured in fresh medium without the compound for several days (1 + *n*). Every third day the medium was replaced by a fresh one. Control cells were treated with dimethyl sulfoxide (DMSO) (Sigma-Aldrich, D4540) in appropriate dose and time.

### Cell transfection

Cells were transiently transfected with mCherry-GFP-LC3B, or mRFP-LC3B and GFP-LAMP-1 constructs using Lipofectamine 2000 (Invitrogen, 11668-019) as described by the manufacturer. Briefly, fifty thousands of cells were transfected with 0.5 µg of mCherry-GFP-LC3B or mRFP-LC3B and GFP-LAMP-1 plasmid and cultured in growth media containing 50 µg/ml neomycin (Sigma-Aldrich, N1142) with exchange of medium every 24 h. 2 days after transfection cells were used for treatments and confocal imaging.

### Cell viability estimation (MTT and Trypan Blue assay)

3-(4,5-Dimethylthiazol-2-yl)-2,5-diphenyltetrazolium bromide (MTT) solution (5 mg/ml) (Sigma-Aldrich, M5655) was added to cells treated for 24 h with the indicated concentrations of C10. Cells were then incubated for 2 h at 37 °C in a humidified atmosphere (5% CO_2_). Formazan formed in living cells was dissolved in DMSO and absorbance of the solution was measured at 570 nm using a microplate reader (Reader 400 SFC, LabInstruments).

Cell viability was also assessed by trypan blue exclusion assay. After treatment with C10, the cells were collected by trypsinization, incubated with Trypan Blue (Sigma-Aldrich, T8154) and viable cells as well as dead ones (blue) were counted.

### Cell death detection

After treatment with the indicated concentrations of C10 cells were washed with phosphate buffer saline (PBS) and stained with 7-amino-actinomycin D (7-AAD) (BD Biosciences, 51-68981E) for 20 min at room temperature (RT). Flow cytometry analyses were performed using FACSCalibur (BD) and the CellQuest analysis software (BD); 10,000 events were counted per sample.

### Bromodeoxyuridine incorporation assay

For DNA synthesis assay, bromodeoxyuridine (BrdU) (10 μM) (Sigma–Aldrich) was added to the medium and cells were cultured for 24 h. BrdU was detected using primary mouse antibody against BrdU (10 µl/ml) (Becton Dickinson) and anti-mouse Alexa 488 (1:500) (Life Technologies) secondary antibody. Cell nuclei were stained with DAPI. Cells were observed under a Nikon Eclipse 50i microscope with a 40 × /0.75 Nikon lens and the ImagePro Plus.

### AChE activity assay

To measure AChE activity the Acetylcholinesterase Assay Kit (Colorimetric) (Abcam, ab138871), which is based on the method of Ellman^56^, was used. The samples for analysis were obtained by sonication of MCF-7 cells treated with IC_50_ dose of C10 or incubation of sonicated control cells with IC_50_ dose of C10. The samples of human erythrocyte AChE were obtained as described by Zawadzka et al.^28^.

### DNA content and granularity analysis

After treatment with IC_50_ dose of C10 for 24 h the medium was discarded and cells were collected by trypsinization and fixed in 70% ethanol. For DNA analysis, cells were stained with solution of propidium iodide (PI) in PBS (3.8 mM sodium citrate, 500 μg/ml RNAse A, 50 μg/ml PI). Granularity and DNA content was determined by flow cytometry using the CellQuest Software; 10,000 events were counted for each sample (FACSCalibur, Becton Dickinson).

### Western blotting analysis

Whole cell protein extracts were prepared according to the Laemmli method^57^. Equal amounts of protein were separated electrophoretically in 10 or 12% SDS-polyacrylamide gels and transferred to nitrocellulose membranes. Membranes were blocked with 5% non-fat dry milk dissolved in TBS containing 0.1% Tween 20 for 1 h at RT and incubated overnight at 4 °C with one of the primary antibodies (in TBS + 0.1% Tween 20/5% milk): rabbit anti-AChE, mouse anti-p21, mouse anti-p62, rabbit anti-ATG5, rabbit anti-LC3B, rabbit anti-AKT (phospho Ser473), rabbit anti-AKT, rabbit anti-p70S6K (phospho Thr389) and mouse anti-β-ACTIN or mouse anti-GAPDH as a loading control. Specific proteins were detected after 1 h of incubation at RT with appropriate horseradish peroxidase–conjugated secondary antibody (1:2000 in TBS + 0.1% Tween 20/5% milk). The ECL system (GE Health Care, #34080) was used according to the manufacturer’s instruction. All proteins were analyzed by Western blotting at least 3 times (3 independent experiments).

### Detection of Senescence-Associated-β-galactosidase

Detection of Senescence-Associated-β-galactosidase (SA-β-gal) was performed according to Dimri *et al*.^58^. Briefly, cells were fixed in 2% formaldehyde, 0.2% glutaraldehyde in PBS, washed, and incubated overnight at 37 °C with a solution containing 1 mg/ml 5-bromo-4-chloro-3-indolyl-β-d-galactopyranoside, 2.5 mM potassium ferrocyanide, 2.5 mM potassium ferricyanide, 150 mM NaCl, 2 mM MgCl_2_ and 0.1 M phosphate buffer, pH 6. Cell nuclei were stained with DAPI (1 µM in PBS) (Sigma-Aldrich, D9542). Cells were observed under a Nikon Eclipse 50i microscope with a 40 × / 0.75 Nikon lens and the ImagePro Plus.

### Cytokine measurement

Conditioned medium was collected at appropriate time points then frozen at −80 and kept until cytokine measurement. Cytokines—IL-6 and IL-8, were estimated using a BD LSRFortessa flow cytometer, equipped with 488 nm and 640 nm lasers and BD FACSDiva 6.2 software. Beads fluorescence was detected with 575/26, 670/14 and 780/60 band passes (BPs) and gated according to the Cytometric Bead Array (CBA) Human Enhanced Sensitivity Master Buffer Kit manual. Data were analyzed with BD FCAP Array TM 3.0 software.

### Immunocytochemistry

Cells grown on coverslips were fixed in 4% paraformaldehyde (PFA) (Sigma-Aldrich, P6148) for 15 min at RT. Then, cells were washed twice with PBS and blocked with 5% bovine serum albumin (BSA) (Sigma-Aldrich, A2153) in PBS containing 0.1% Triton X-100 for 30 min. After washing cells were incubated with appropriate antibody diluted in PBS + 0.1% Triton X-100/1% BSA—first with primary rabbit anti-LC3B monoclonal antibody or mouse anti-ATG16L antibody for 2 h and next with anti-rabbit Alexa 488 or anti-mouse Alexa 555 secondary antibody for 1 h. DNA was stained with DAPI (1 µM in PBS) and the coverslips were mounted. LC3B and ATG16L foci were visualized with Nikon Eclipse Ti, a fluorescent microscope with a 40 × /0.6 Nikon lens, and analyzed using the NIS Elements Basic Research software.

### Electron microscopy

Glutaraldehyde was added to cells collected by trypsinization to the final concentration of 2%. Cells were pelleted (conditions: 163 × g, 5 min) and fixed for 1 h in 4 °C. After fixation, cells were rinsed two times for 10 min in 0.1 M cacodylate buffer, then incubated overnight in 4 °C. Next, cell pellets were postfixed in 1% osmium tetroxide for 1 h at RT. Dehydration was performed by incubating the sample in increasing ethanol concentrations and in pure propylene oxide. During dehydration cells were stained with 1% uranyl acetate in 70% ethanol. Finally, cells were embedded in the Epon resin. Sixty-five nanometer sections were prepared and poststained with uranyl acetate and Reynold’s lead citrate. Photos were obtained with Morada camera on a JEM 1400 transmission electron microscope at 80 kV (JEOL Co., Japan).

### Quantitative analysis

Computational analysis of cell number, foci number, area occupied by autophagic vesicles and Pearson’s correlation coefficient for protein colocalization was determined using ImageJ (FiJi) software. More than 100 cells were counted per sample for each analysis except for estimation of the area occupied by autophagic vesicles from electron microscopy images (30 cells per sample).

### Statistical analysis

All experimental results are presented as means ± SD except when indicated otherwise. Statistical analysis was conducted using the STATISTICA software. Two group comparisons were performed using the Mann–Whitney test. A *p* value of <0.05 was considered as statistically significant. Asterisks denote *p < 0.05, **p < 0.01, ***p < 0.001.

## Electronic supplementary material

Supplemental Figure 1

Supplemental Figure 2
