# Supplementary material for: Simultaneous induction and blockade of autophagy by a single agent
Source: Cell Death Dis. 2018 Mar 2;9(3):353. doi: 10.1038/s41419-018-0383-6 (PMC5834631; doi:10.1038/s41419-018-0383-6)
Supplement: Supplementary file 1 — Supplemental Figure 1 [file 41419_2018_383_MOESM1_ESM.pdf]

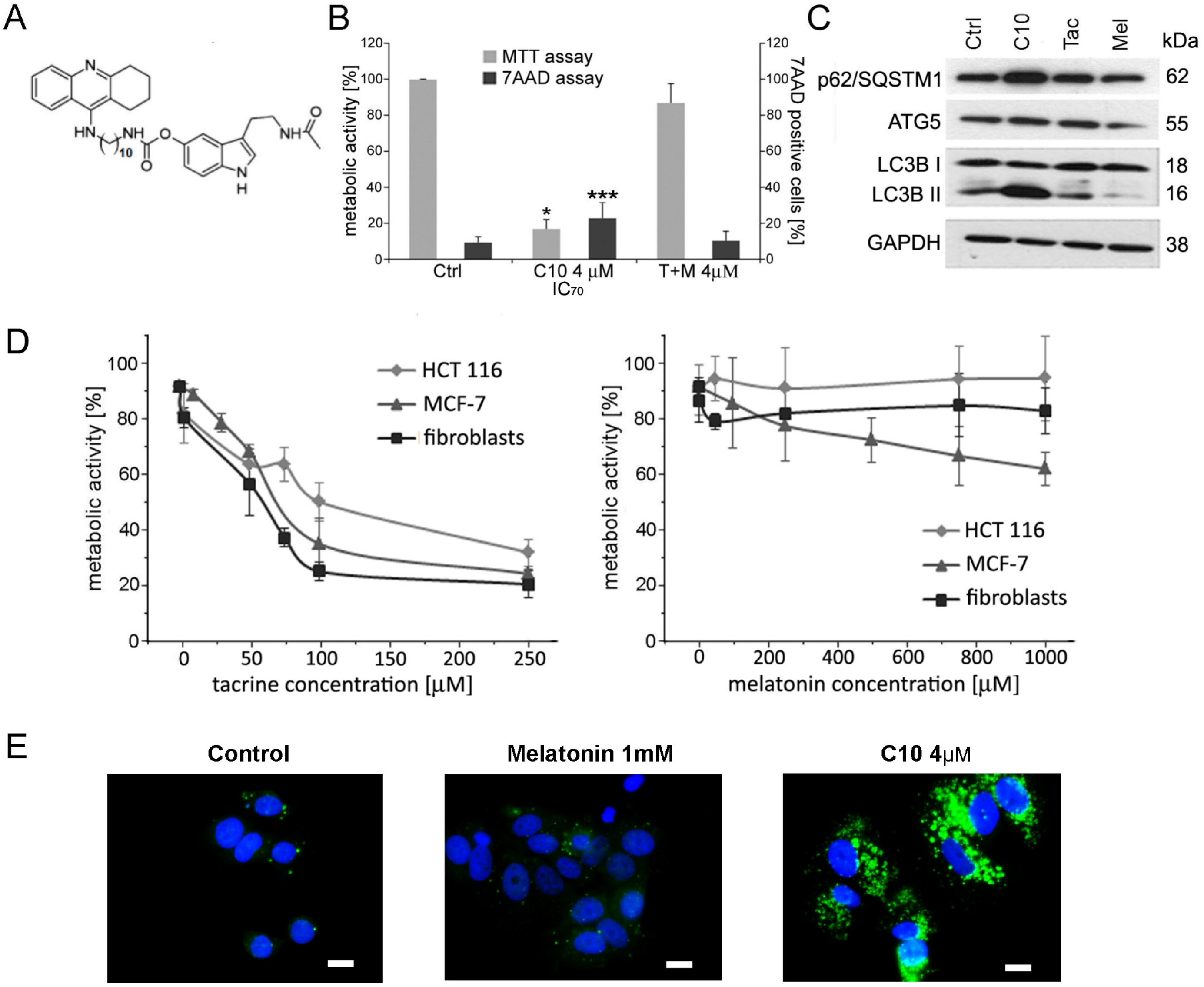

Supplemental Figure 1. Cytostatic/cytotoxic effect and autophagy modulation by heterodimer C10 but not its components. (A) Formula of heterodimer C10. (B) Viability of MCF-7 cells treated for one day with 4 µM C10 or with both 4 µM tacrine and 4 µM melatonin (T+M) measured by MTT assay (6 independent experiments) and 7AAD assay (5 independent experiments). Data was calculated as the percentage of control or percentage of total cell population for MTT and 7AAD assay, respectively. (C) Representative western blot of p62/SQSTM1, ATG5 and LC3B protein level in control cells and cells treated with IC_{50} dose of C10 (3 µM), 3 µM tacrine (Tac) or 3 µM melatonin (Mel) for one day. (D) Viability of MCF-7, HCT 116 cells and fibroblasts, treated for 24 h with indicated concentrations of tacrine or melatonin, measured by MTT assay (3 independent experiments). (E) Representative immunofluorescence images of LC3B (green) and nuclei stained with DAPI (blue) in MCF-7 cells treated for 24h with the indicated compound. Scale bars: 20 µm.
