# Supplementary material for: Simultaneous induction and blockade of autophagy by a single agent
Source: Cell Death Dis. 2018 Mar 2;9(3):353. doi: 10.1038/s41419-018-0383-6 (PMC5834631; doi:10.1038/s41419-018-0383-6)
Supplement: Supplementary file 2 — Supplemental Figure 2 [file 41419_2018_383_MOESM2_ESM.pdf]

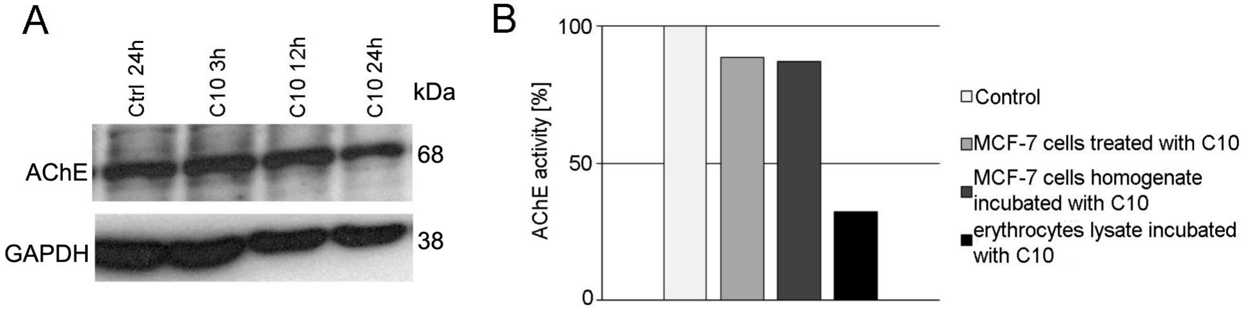

Supplemental Figure 2. AChE protein level and activity in MCF-7 cells after C10 treatment.
(A) Representative western blot of AChE protein level in control MCF-7 cells and treated with C10 for the indicated time.
(B) AChE activity in MCF-7 cells, homogenate of these cells or erythrocyte lysate treated with IC50 dose of C10. Graph represents mean value from 2 independent experiments.
